# Advances, Overuse, and Shared Decision-Making in Abdominal Wall Surgery

**DOI:** 10.3389/jaws.2025.14951

**Published:** 2025-09-04

**Authors:** Manuel López-Cano, Josep M. García-Alamino

**Affiliations:** ^1^ Abdominal Wall Surgery Unit, Hospital Universitaio Vall d´Hebrón, Universidad Autónoma de Barcelona (UAB), Barcelona, Spain; ^2^ Global Health, Gender and Society (GHenderS), Facultat de Ciències de la Salut, Blanquerna-Universitat Ramón Llull, Barcelona, Spain

**Keywords:** hernia, abdominal wall, overuse, overtreatment, shared decision making

## Introduction

Abdominal wall hernia repair has progressed from suture‐based open techniques to mesh-reinforced open repair, laparoscopic intraperitoneal onlay mesh (IPOM), endoscopic retromuscular approaches and most recently robot-assisted reconstructions [1]. Of course, this trajectory has provided important benefits (smaller incisions, faster recovery and best anatomical reconstructions and dissections) but it has also brought some drawbacks (rising procedure volumes, cost inflation, and treatment of hernias that might remain asymptomatic for years) [2, 3]. That phenomenon (providing care whose potential benefit is eclipsed by its opportunity cost or risk) is labelled low-value care [4]. In abdominal wall surgery the line between value and overuse can sometimes be blurred and difficult to identify because most ventral, incisional and inguinal hernias are elective, their natural history variable, and the evidence base irregular.

The purpose of this article is to make a reflection about the progressive evolution of abdominal wall surgery, the emergence of overuse as a major risk, the drivers behind low-value care and why shared decision making (SDM) (particularly the “option-talk” phase that weighs among other things the data efficacy and safety) may be one of the best antidote. A brief point of view is also made about the quality of the evidence surgeons rely on, including an explosion of publications and concerns that conventional peer review may be struggling which can hinder disruptive findings. Finally, it offers a focused reflection on robotic abdominal wall surgery, highlighting weak randomised data, divergent incentives between clinicians and industry and the argument for slowing the adoption curve.

## Technical Evolution of Abdominal Wall Surgery

Early open herniorrhaphy required large incisions, prolonged convalescence and high recurrence rates [1]. Tension-free mesh repair reduced recurrence dramatically. Laparoscopic intraperitoneal mesh placement (IPOM) in the 1990s further lowered wound morbidity and hastened return to work. Over the last decade endoscopic retromuscular techniques such as eTEP and eTAR have recreated the biomechanical advantages of open sublay repair without a laparotomy [5]. Parallel progress in biomaterials has refined the balance between strength and foreign-body response [6]. Most recently, the robotic platform has extended wristed instrumentation and 3-D vision to hernia surgery allowing for improved minimally invasive approach for complex cases [7]. Artificial-intelligence image guidance and risk-prediction tools are now entering the field [8].

## Overuse and Low-Value Care

Globally over 20 million groin and over 700,000 ventral hernias are repaired each year [9]. Population studies show wide geographic variation in elective repair rates that cannot be explained by prevalence alone a classic signal of overuse [10]. In high income settings fee-for-service payment, defensive medicine, direct-to-consumer marketing, surgeons’ learning-curve incentives and patients’ expectations (“fix it now”) all propel intervention [11]. In low and middle-income countries different mechanisms appear, e.g., donor funding that prioritises volume rather than need, or import-driven dependence on premium meshes even where low-cost alternatives perform similarly [12, 13]. The harm could be two-fold: individually, unnecessary repairs expose patients to mesh infection, chronic pain and loss of work without offsetting benefit; systemically, they divert theatre time and budgets from higher-value services.

## Shared Decision Making

SDM reframes surgical consent from a one-way recommendation to an iterative conversation: choice talk (there is more than one path), option talk (weighing pros and cons), and decision talk (aligning with patient values). Randomised and observational studies in abdominal wall surgery show that decision aids reduce surgery rates without harming quality of life and increase preference-concordant care [14–16]. For us, option talk plays a key role in the SDM process and the *option talk* is only as good as the evidence on efficacy and safety that underpins it. Whether individual studies and/or systematic reviews are methodologically weak or skewed by publication bias the risk–benefit picture patients receive is distorted potentially fuelling overuse rather than curbing it.

## Evidence Under Strain: Quantity Vs. Quality

The volume of surgical literature grows around 5% per year doubling every 14–17 years [17, 18]. Peer-review pipelines and editorial standards have not scaled at the same pace prompting concern that “special-issue” models and article-processing-charge incentives could erode quality control [19, 20]. Bibliometric studies show that while citations and team sizes soar the proportion of truly disruptive papers (those that redirect a field) has fallen sharply since the mid-1940s [20]. Critics argue that review burden, risk-averse funding and industry partnerships favour incremental work at the expense of bold ideas, leaving surgeons with an avalanche of low-signal data [21]. For abdominal wall surgery it could mean hundreds of small retrospective series and few adequately powered trials. Surgeons therefore rely on evidence that may be internally valid but externally fragile, again jeopardising option talk in SDM process.

## The Robotic Spotlight

Robotic hernia repair (RHR) provides ergonomic suturing and easier retro-rectus dissection. Systematic reviews of observational studies suggested lower wound morbidity and shorter length of stay [22]. However, the first prospective randomised controlled trial comparing robotic with open retromuscular repair found no difference in composite surgical-site morbidity or 2-year recurrence, although length of hospital stay was 1 day shorter with robotics [23]. A registry-based multicentre RCT (ROVHR) is under way but still recruiting [24]. Meta-analyses report similar outcomes but consistently higher direct theatre costs for RHR [25]. Device amortisation, longer set-up time, and disposable instrument pricing shift the cost-effectiveness threshold unfavourably when clinical benefit is marginal. Yet adoption curves remain steep, driven by marketing, institutional prestige, and surgeon training pathways aligned with robotic consoles. Surveys indicate that trainees perceive robotics as the default future despite uncertain evidence, could it be a sign of for technology-driven overuse? [26, 27]. In addition, it may be that in today’s abdominal wall surgery exercice the traditional dogma associated with individual practice (study, research, publication, and evidence-based acquisition of technical skills) may be challenged and replaced by other influences associated with opinion leaders, practice conformity, and reputational concerns [28].

## Integrating Trials Into Practice

The translation gap after surgical trials is often blamed on heterogeneity of technique and learning curves, but financial incentives also matter [29, 30]. Manufacturers underwrite proctoring and conference symposia, whereas public funders bankroll the few head-to-head trials that might curb sales. Clinicians, under time pressure and mindful of competitive advantage may prioritise early adoption over equipoise. This divergence creates what some have called a “house divided”: clinical equipoise versus commercial imperatives because clinical trials and healthcare delivery companies could be assisted by separate people, policies, institutions, and funding, leading to different motivations and aims [31, 32]. Registries and audit networks offer a partial bridge, but without comprehensive cost data even robust outcome tracking cannot answer value questions [33]. Slow-medicine and surgery advocates [34, 35] could argue that abdominal-wall surgery should take a lesson and insist on large, transparent, multi-stakeholder trials before population-level diffusion, in other words, modernizing the data infrastructure for clinical research avoiding postmarketing analysis [32]. Until then, SDM remains the ethical buffer allowing patients to choose or decline new technologies, devices or surgical techniques based on the current imperfect knowledge.

## Discussion

The abdominal wall surgery exemplifies the surgical innovation of the XXI century with an unstoppable and fast evolution that seems to have immersed the surgeon interested in this pathology in a constant spiral of innovations and new concepts. However, knowledge (and the patient in the clinic) move at human speed. Low-value care emerges where that mismatch increases. Our opinión article could highlight four possible strategic responses. First, deepen the evidence base: pragmatic, registry-embedded RCTs should become the norm, with mandatory cost-utility endpoints. Publishers and funders must reward disruptive designs and negative trials alike. Second, slow premature diffusion: health systems can condition procurement of new devices or technological platforms on demonstrable incremental benefit not prestige. Third, operationalise SDM: decision aids, visual risk formats and “option talk” should be embedded in hernia pathways and audited like any other quality metric. Finally, realign incentives: bundled payments, appropriateness criteria and public reporting of long-term outcomes counteract volume-based drivers of overuse.

The field is unlikely to revert to pre-mesh open repairs nor should it. But progress that outpaces reflection ceases to be progress. A deliberate pause (time to study, synthesise and decide with patients) may feel alien in an era of technological exuberance yet it is precisely the antidote to low-value care [20]. Abdominal wall surgery is not a race to implant the most advanced mesh with the newest technological platform it is a commitment to restore abdominal core function safely, effectively and only when truly necessary.
